# Concurrent Inguinal Endometriosis and Catamenial Pneumothorax: A Case Report

**DOI:** 10.7759/cureus.78747

**Published:** 2025-02-08

**Authors:** Yu Tanaka, Naoki Horikawa, Tomoki Nishimura, Hikaru Kiyokawa, Ken Fukuhara

**Affiliations:** 1 Department of Obstetrics and Gynecology, Kurashiki Central Hospital, Kurashiki, JPN; 2 Department of Obstetrics and Gynecology, Hamamatsu University School of Medicine, Hamamatsu, JPN

**Keywords:** catamenial pneumothorax, endometriosis, extra-pelvic endometriosis, inguinal endometriosis, intrathoracic endometriosis, laparoscopic surgery, video-assisted thoracoscopic surgery (vats)

## Abstract

Endometriosis, while prevalent, can manifest in extra-pelvic locations with varying degrees of rarity, but reports of multiple extra-pelvic sites within a patient are extremely rare. We report a unique case of a 45-year-old female with concurrent inguinal endometriosis and catamenial pneumothorax. The patient experienced recurrent menstruation-associated right chest pain and subsequently developed right inguinal pain. A laparoscopic-assisted en bloc resection of the round ligament and associated inguinal mass was performed. Intraoperative findings were consistent with pelvic endometriosis. Hormonal therapy was not initiated due to the patient desiring pregnancy, but despite assisted reproductive technology, pregnancy was unsuccessful. Subsequent investigation following the onset of right chest pain and dyspnea revealed right pneumothorax. Thoracoscopic intervention identified diaphragmatic defects suggestive of endometriosis. Resection of the lung parenchyma with an air leak, along with suture repair of a diaphragmatic defect, was performed. Postoperatively, the pneumothorax resolved. Post-surgical hormonal therapy with dienogest resulted in the resolution of both conditions. This case underscores the potential for diverse presentations of extra-pelvic endometriosis and highlights the importance of a multidisciplinary approach to its management.

## Introduction

Endometriosis is a gynecological condition characterized by the presence of endometrial-like tissue outside the uterine cavity, most commonly found in the ovaries, fallopian tubes, and pelvic peritoneum [1]. These ectopic endometrial tissues respond to hormonal fluctuations, leading to cyclic growth, shedding, and subsequent inflammation and scarring [2], often resulting in symptoms such as dysmenorrhea, chronic pelvic pain, dyspareunia, dyschezia, and infertility [1]. It is estimated that approximately 10% of women of reproductive age, which equates to about 190 million women worldwide, are affected by this disease. Moreover, endometriosis can have a significant economic impact. In the United States, the annual medical cost per patient with endometriosis was estimated to be $4,000 in 2008, comparable to chronic conditions such as type 2 diabetes, Crohn's disease, and rheumatoid arthritis [1]. The decreased quality of life experienced by women with endometriosis can also lead to significant societal costs. While the exact etiology remains elusive, proposed mechanisms include retrograde menstruation [3], coelomic metaplasia [1], and lymphatic or hematogenous dissemination [4,5]. 

Typically confined to the pelvis, endometriosis can manifest extra-pelvically in various organs, including the intestines, urinary tract, thorax, and abdominal wall. Extra-pelvic endometriosis can cause diverse and potentially life-threatening complications, such as catamenial pneumothorax [6], bowel obstruction [7], and urinary tract obstruction [8]. Due to its rarity, extragenital endometriosis is often diagnosed late, particularly when it presents in organs outside the reproductive system. This delay in diagnosis can be attributed to the fact that physicians in specialties other than gynecology may not consider endometriosis in their differential diagnosis. Furthermore, the lack of standardized treatment protocols for extragenital endometriosis can make management challenging and can significantly impact a patient's quality of life.

Extra-pelvic endometriosis often coexists with pelvic endometriosis [9], and the presence of pelvic endometriosis can serve as a clue to the diagnosis of extra-pelvic endometriosis. However, the simultaneous occurrence of endometriosis in multiple extra-pelvic sites within a single patient is extremely rare. This case report presents a unique patient who experienced a 14-year clinical course characterized by the initial onset and spontaneous resolution of catamenial pneumothorax, followed by the diagnosis and treatment of inguinal endometriosis, subsequent recurrence of catamenial pneumothorax, and successful hormonal therapy. This case highlights the complex nature of endometriosis, the importance of surgical intervention, and the role of hormonal therapy in preventing recurrence, offering insights into the pathogenesis of extra-pelvic endometriosis.

This case was previously presented as a meeting abstract at the 46th Annual Meeting of the Endometriosis Society of Japan on January 26, 2025.

## Case presentation

**Figure 1:**
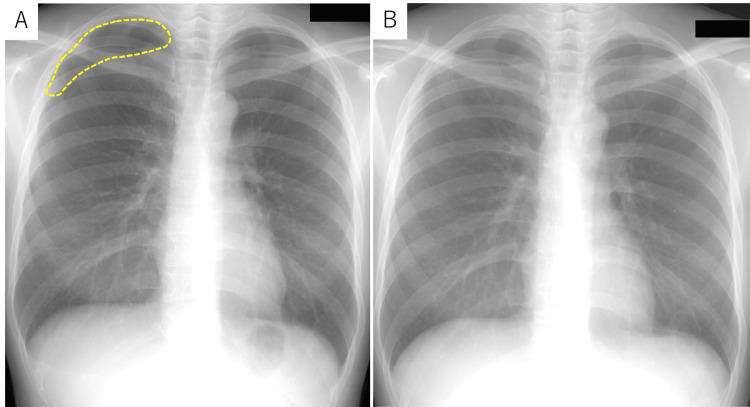
(A) A small pneumothorax is noted in the right hemithorax, primarily involving the right upper lobe (demarcated by a yellow dotted line). (B) Complete resolution of the right pneumothorax is evident on the follow-up chest X-ray obtained eight days later.

**Figure 2:**
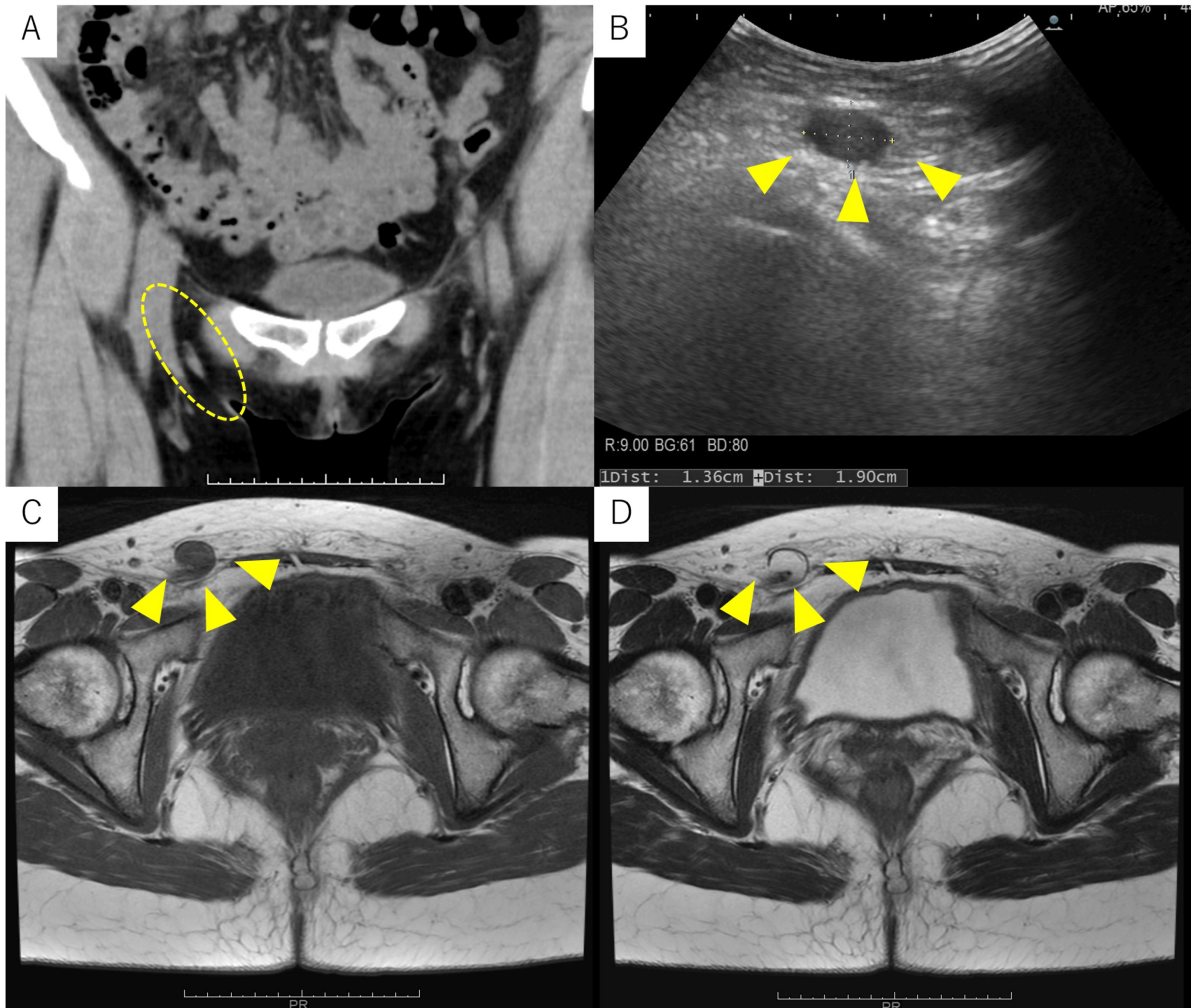
(A) CT imaging showed slight swelling of the right inguinal canal compared to the contralateral side (yellow dotted oval), raising the possibility of inguinal endometriosis. (B) Ultrasonography of the right inguinal region revealed a 1.36 x 1.90 cm subcutaneous mass (yellow arrowheads). (C) T1-weighted MRI revealed a structure exhibiting intermediate to low signal intensity within the right inguinal canal (yellow arrowheads). (D) T2-weighted MRI revealed a structure exhibiting high signal intensity within the right inguinal canal (yellow arrowheads). These findings raised suspicion for endometriosis of the right inguinal region.

A 45-year-old female with a history of one cesarean section presented with a history of recurrent right-sided chest pain that coincided with her menstrual cycles. This history began at the age of 31. At that time, she experienced cyclical right-sided chest pain coinciding with her menstrual cycle. A diagnosis of catamenial pneumothorax was suspected (Figure 1A). However, as symptoms resolved spontaneously (Figure 1B), hormonal and surgical treatments were deferred, and she was advised to seek medical attention if symptoms worsened. At the age of 39, she visited an internal medicine clinic with right inguinal pain as the chief complaint. Ultrasound and computed tomography (CT) scans suggested right inguinal endometriosis, and she was referred to our department (Figure 2A). Magnetic resonance imaging (MRI) revealed right inguinal endometriosis as well as pelvic endometriosis. Hormonal therapy was withheld due to the patient desiring pregnancy. Despite conservative management and subsequent attempts at assisted reproductive technology, pregnancy was unsuccessful. 

**Figure 3:**
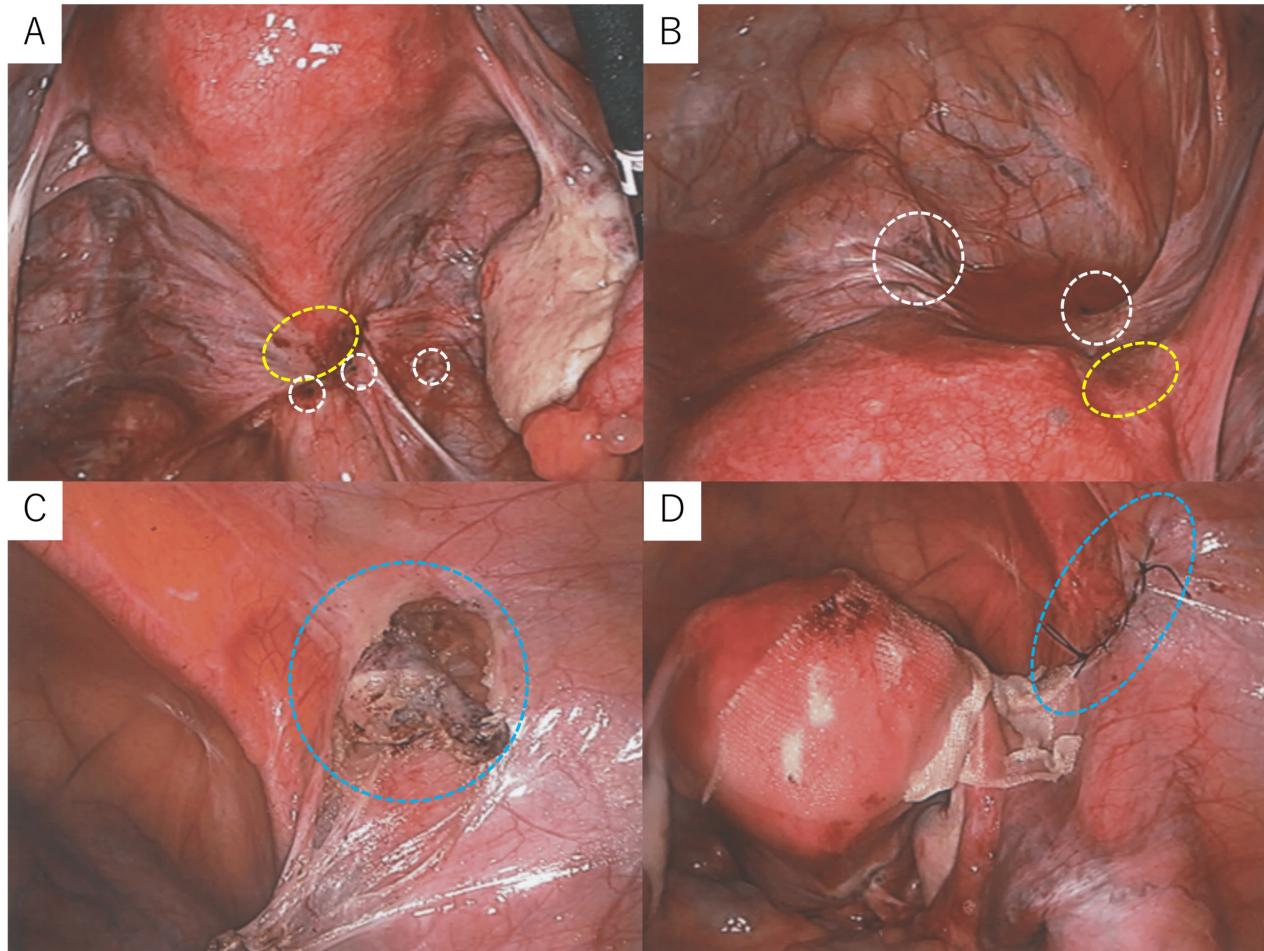
(A) Laparoscopic view of the Douglas' pouch demonstrating peritoneal adhesions with areas of hemorrhage (yellow dotted oval) and pigmentation (white dotted circles). (B) Laparoscopic view of the vesicouterine pouch demonstrating peritoneal adhesions with areas of hemorrhage (yellow dotted oval) and pigmentation (white dotted circles). (C) Intraoperative image demonstrating dissection of the right round ligament towards the internal inguinal ring (blue dotted circle). (D) Intraoperative image demonstrating closure of the peritoneal incision made during right round ligament excision (blue dotted oval).

**Figure 4:**
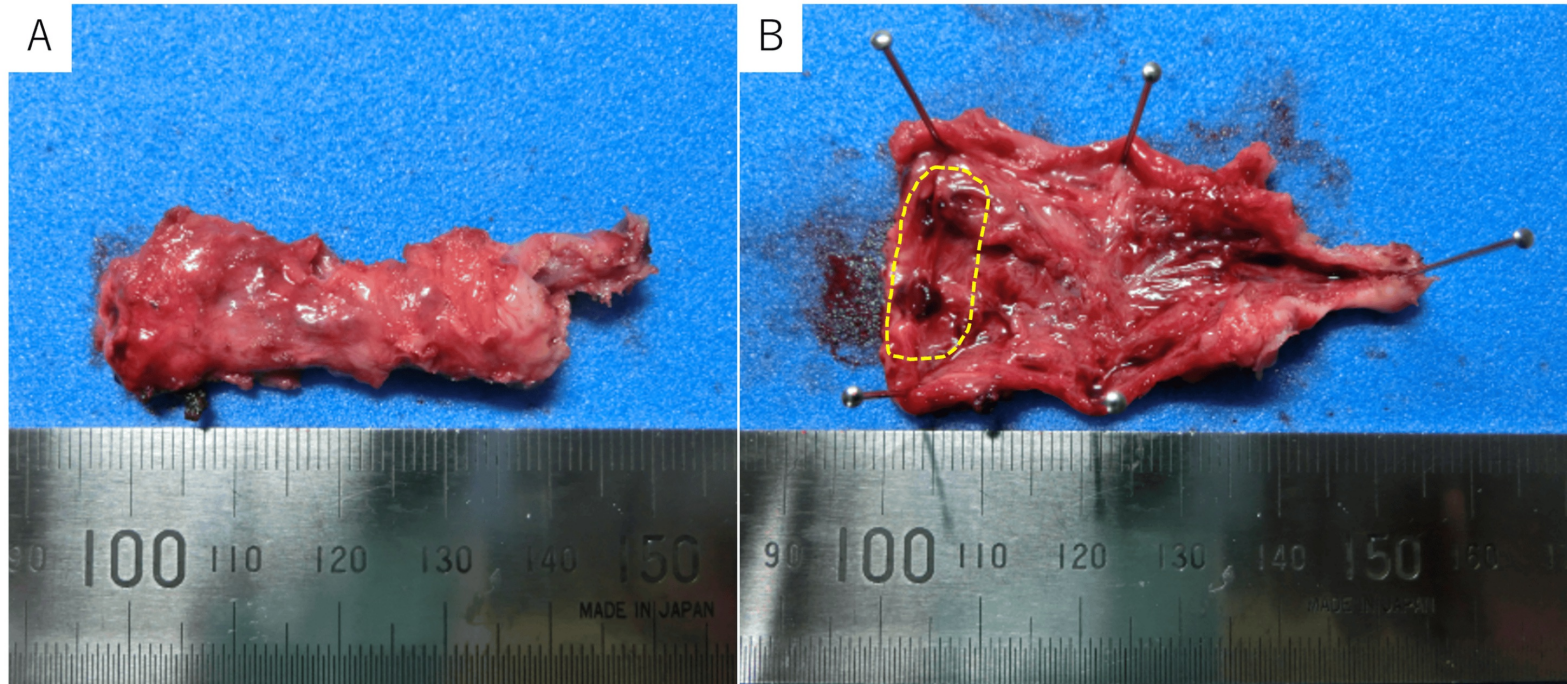
(A) Gross specimen of the excised right round ligament. (B) Sectioning of the excised right round ligament revealed a black, hemorrhagic lesion at the distal end (demarcated by a yellow dotted line).

The patient re-visited our department at age 40 due to exacerbation of right inguinal pain. Ultrasound (Figure 2B) and MRI (Figure 2C, 2D) revealed an increase in the size of the right inguinal endometriosis, which prompted laparoscopic-assisted right round ligament resection and inguinal hernia repair, performed in collaboration with the surgical department. The Douglas' pouch was obliterated by adhesions due to endometriosis. Scattered red and black lesions were observed in the Douglas' pouch, uterine surface, and vesicouterine pouch, consistent with stage II (eight points) pelvic endometriosis according to the revised American Society for Reproductive Medicine (rASRM) classification (Figure 3A, 3B). A uterine manipulator was inserted vaginally, and the right round ligament was dissected 1.5 cm from the uterus using an ultrasonic scalpel (Figure 3C). The broad ligament was incised to isolate the round ligament to the internal inguinal ring. Subsequently, the skin over the right groin was incised, the inguinal canal was opened, and the right round ligament was excised en bloc (Figure 4A, 4B). The widened internal inguinal ring was sutured. Laparoscopic manipulation was resumed, and the open peritoneum near the internal inguinal ring was sutured and closed. An adhesion barrier was applied to the uterine corpus from the round ligament stump, and the laparoscopic procedure was completed (Figure 3D). Pathological examination of the excised specimen revealed endometriosis. Postoperative course was uneventful, and right inguinal pain disappeared. Despite resumption of assisted reproductive technology, pregnancy was unsuccessful and the patient discontinued follow-up visits.

**Figure 5:**
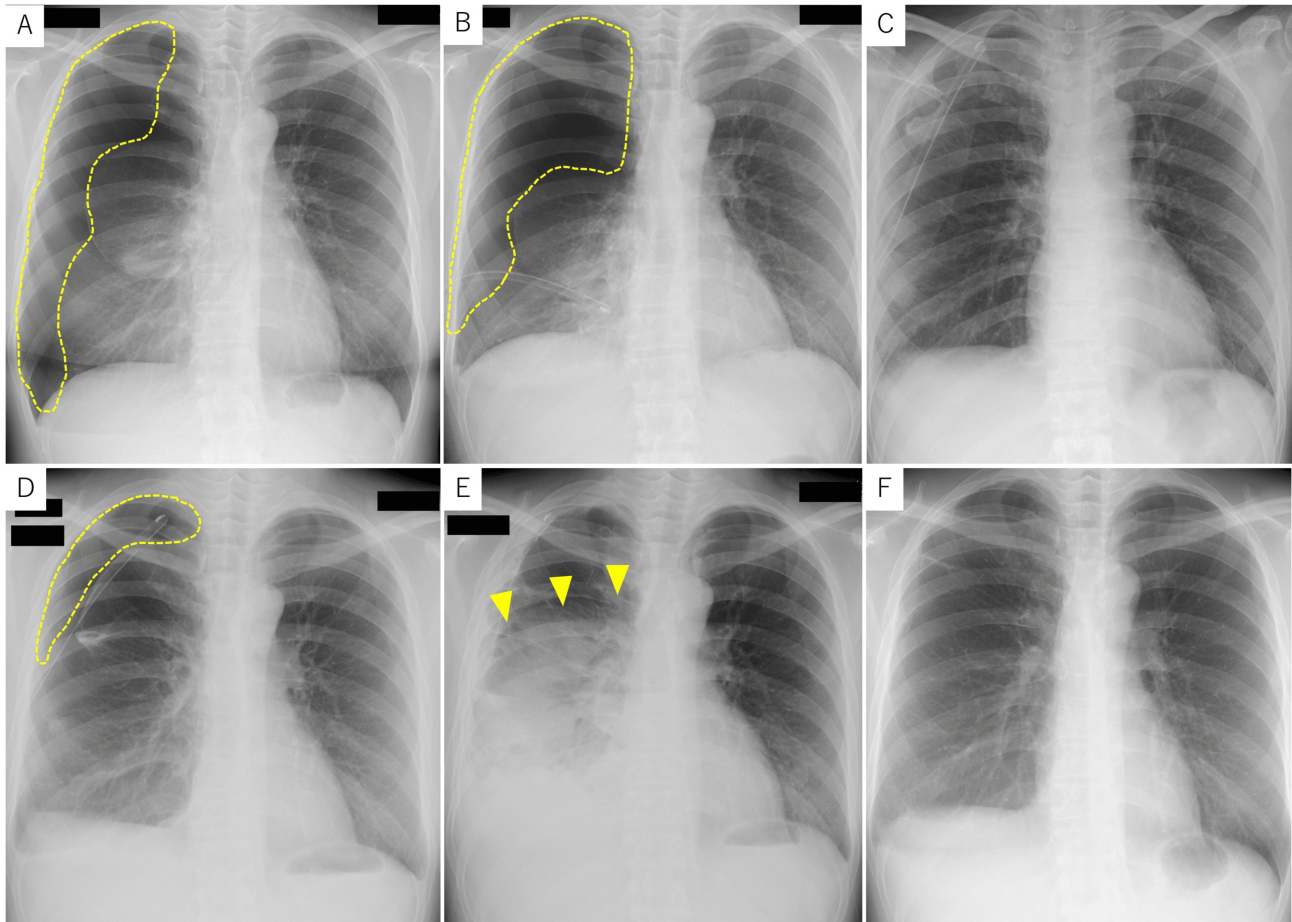
(A) Chest radiograph demonstrating right lung collapse consistent with pneumothorax (demarcated by a yellow dotted line). (B) Despite chest tube placement, the right lung collapse progressed (demarcated by a yellow dotted line). (C) Postoperative chest radiograph obtained on the following day demonstrated satisfactory lung expansion. (D) On postoperative day three, chest radiography revealed inadequate expansion of the right lung (demarcated by a yellow dotted line). (E) Postoperative day 13 chest radiograph revealed right pleural effusion (yellow arrowheads); however, aeration of the right lung had improved following three pleurodesis procedures. (F) Chest radiograph obtained 2.5 years post-thoracotomy for pneumothorax revealed no evidence of pulmonary collapse.

**Figure 6:**
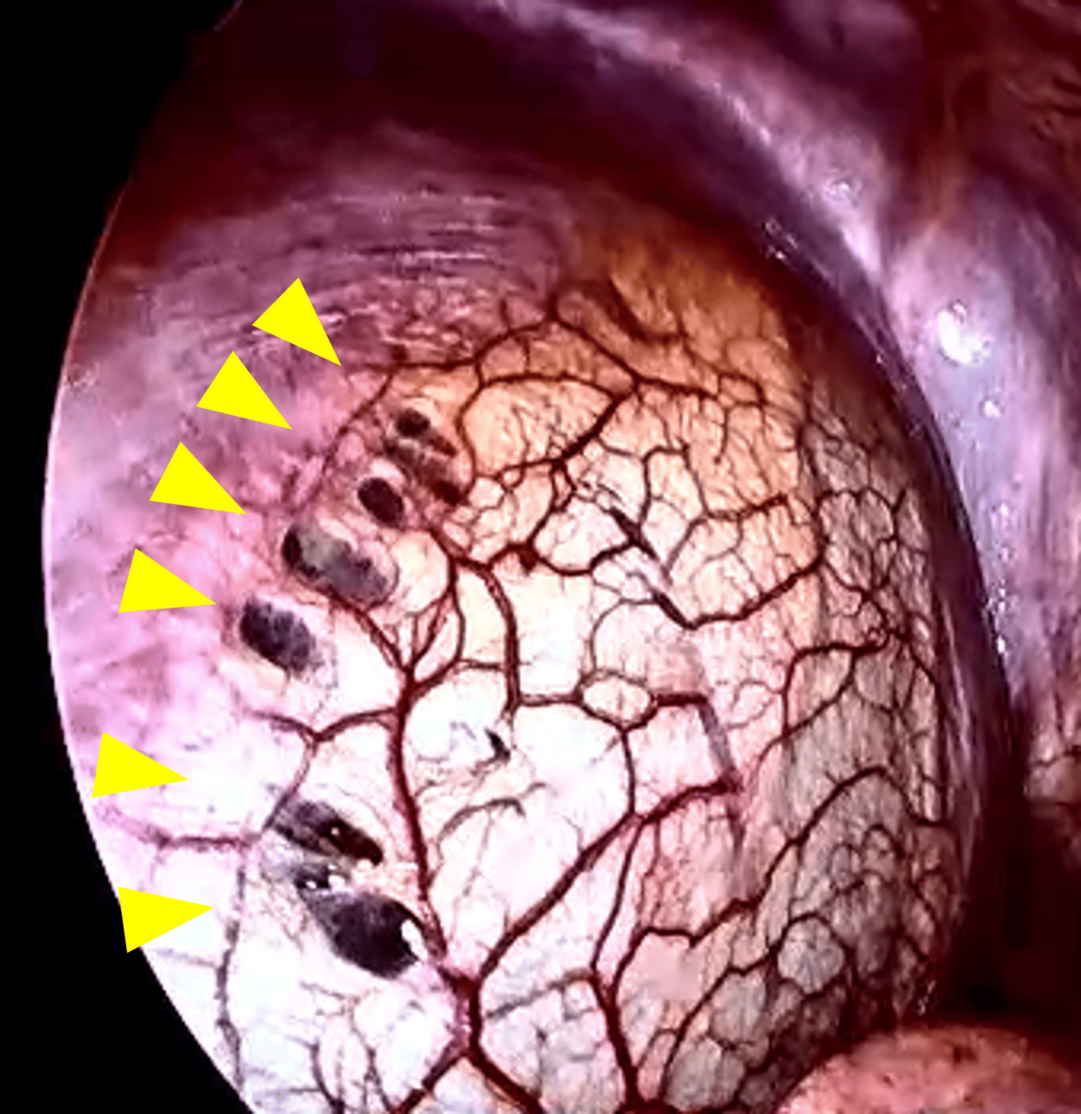
Thoracoscopic findings demonstrating multiple perforations (yellow arrowheads) in the right hemidiaphragm, suggestive of endometriosis.

At around the age of 40, she began experiencing recurrent right-sided chest pain every few months. Due to the self-limiting nature of these episodes, she did not seek medical attention. At the age of 43, the patient presented to the emergency department with recurrent right-sided chest pain and dyspnea that had worsened and failed to resolve spontaneously, prompting her to seek medical attention. Chest X-ray revealed right pneumothorax (Figure 5A), and she was urgently admitted to the thoracic surgery department. Despite conservative management of pneumothorax with chest tube placement, persistent air leak prevented adequate lung re-expansion (Figure 5B). Video-assisted thoracoscopic surgery (VATS) was performed to identify and resect the underlying cause of the air leak. No obvious bullae were observed, but air leaks from the right upper lobe and multiple small perforations in the right hemidiaphragm suggested endometriosis (Figure 6). A partial lobectomy of the right upper lobe was performed to address the persistent air leak. A small diaphragmatic defect was identified and sutured closed. Postoperatively, lung expansion was initially satisfactory (Figure 5C), but an air leak developed, leading to incomplete lung re-expansion (Figure 5D). Despite continued chest tube drainage, the air leak persisted, necessitating three additional pleurodesis procedures on postoperative days five, nine, and 12. The air leak resolved after the final procedure (Figure 5E). Histological assessment of the resected lung tissue did not definitively establish the diagnosis of endometriosis, but medical history and thoracoscopic findings led to the clinical diagnosis of intrathoracic endometriosis. Postoperatively, the patient was initiated on leuprolide acetate, a gonadotropin-releasing hormone (GnRH) agonist, at a dose of 1.88 mg administered subcutaneously every four weeks to induce a pseudo-menopause state. This treatment was continued for six months, followed by a switch to oral dienogest 1 mg twice daily. The patient remains on dienogest to date and has remained asymptomatic with no recurrence of inguinal pain or chest pain (Figure 5F).

## Discussion

Inguinal endometriosis is a rare extrapelvic manifestation of endometriosis, with an estimated prevalence of 0.3-0.6% of all endometriosis cases [10]. Patients typically present with a tender inguinal mass, which can be challenging to differentiate from inguinal hernia or lymphadenopathy. While ultrasound and CT scans can provide valuable information, MRI is often considered the gold standard for diagnosis due to its ability to visualize internal blood components within the mass [10]. In this case, MRI demonstrated a fluid-fluid level on T2-weighted images and slightly higher signal intensity than the bladder on T1-weighted images, strongly suggesting the presence of blood products and supporting the diagnosis of inguinal endometriosis (Figure 2C, 2D).

Surgical excision is generally considered the first-line treatment for inguinal endometriosis, although the rarity of this condition limits the evidence for any specific surgical approach [6]. Due to the high rate of misdiagnosis and the potential for recurrence, a multidisciplinary approach involving both gynecology and general surgery is crucial. Many cases of inguinal endometriosis are initially misdiagnosed as inguinal hernias and managed by general surgeons. Subsequent recurrence of inguinal pain and a delayed diagnosis of inguinal endometriosis have been reported in such cases, highlighting the importance of considering endometriosis in the differential diagnosis of inguinal masses [11]. A combined laparoscopic and open approach, involving laparoscopic dissection of the round ligament followed by open excision of the mass and hernia repair if necessary, has become a common surgical approach for inguinal endometriosis [10,12]. This technique allows for simultaneous evaluation of pelvic endometriosis and complete excision of the inguinal mass. While there is currently no definitive evidence supporting the use of hormonal therapy to prevent recurrence, it may be considered for patients with concomitant pelvic endometriosis, especially if there are no contraindications [6]. For women of reproductive age presenting with a painful inguinal mass, imaging studies such as ultrasound, CT, and MRI should be considered. If inguinal endometriosis cannot be ruled out, a multidisciplinary approach involving both gynecology and general surgery should be considered, and referral to a specialized center may be necessary.

Thoracic endometriosis, while rare, is often associated with pelvic endometriosis. The exact prevalence of thoracic endometriosis remains unknown due to its rarity. Thoracic endometriosis can manifest as catamenial pneumothorax, hemothorax, or hemoptysis, with catamenial pneumothorax accounting for 72-73% of cases. Catamenial pneumothorax represents approximately 20-35% of spontaneous pneumothoraces in reproductive-age women [6]. While catamenial pneumothorax is often associated with menstrual cycles, it is important to note that this correlation is not always present. Despite the lack of a clear menstrual link, the diagnosis of endometriosis should be considered in reproductive-age women, especially in cases with recurrent pneumothorax, right-sided predominance, and coexisting infertility. Factors such as cyclical onset coinciding with menstruation or ovulation can further support the diagnosis [13]. VATS is a valuable tool for the diagnosis and treatment of catamenial pneumothorax, allowing for extensive visualization and biopsy of the lung, pleura, and diaphragm, as well as the resection of leaking lung tissue and repair of diaphragmatic defects. Although hormonal therapy has been reported to be effective for catamenial pneumothorax, surgical treatment is considered more effective in preventing recurrence [14]. A combination of surgery followed by hormonal therapy can further reduce the recurrence rate [15]. In 52.1% of cases, VATS findings were diagnostic for catamenial pneumothorax. Common findings included diaphragmatic lesions (38.8%), parietal pleural lesions (29.6%), bullae, blebs, and scars (23.1%) [13]. 

Pathological confirmation of thoracic endometriosis can be challenging due to small lesion size, difficulty in differentiating from inflammation, and the absence of glandular structures; the diagnostic yield has been reported to be between 57% and 87.5% [6]. In the present case, although pulmonary pathology did not reveal endometriosis, typical diaphragmatic defects suggestive of endometriosis were observed during VATS. While the diaphragmatic defect was closed without biopsy, a clinical diagnosis of endometriosis was made, and hormonal therapy was initiated. The patient has remained symptom-free for two years following surgery and hormonal therapy, suggesting that this combined approach was effective. This case highlights the importance of clinical judgment in the diagnosis and management of catamenial pneumothorax, even in the absence of definitive pathological confirmation.

This case report presents a unique clinical course of a patient with concurrent inguinal endometriosis and catamenial pneumothorax. To the best of our knowledge, only four reports have described cases with multiple concurrent extra-pelvic endometriosis, excluding surgical wounds [16-19]. All four previous cases involved catamenial pneumothorax and umbilical endometriosis. We believe this case is the first reported case of inguinal endometriosis and catamenial pneumothorax. Both inguinal endometriosis (75-90%) and catamenial pneumothorax (>90% of cases) preferentially occur on the right side, as with the present case [6,10,12]. Although seemingly disparate, the concurrence of these two rare extra-pelvic endometrioses might share a common mechanism: the hypothesized clockwise flow of peritoneal fluid. It is believed that peritoneal fluid containing endometrial tissue flows from the uterus to the right side which then becomes obstructed by the sigmoid colon. This allows the endometrial tissue to settle in the right round ligament through the right inguinal canal, causing right inguinal endometriosis. Similarly, peritoneal fluid containing endometrial tissue is believed to travel through the right paracolic gutter to the right upper abdomen, which then becomes obstructed by the falciform ligament of the liver. This allows endometrial tissue to settle under the right diaphragm. Intrathoracic endometriosis ensues upon the endometrial tissue entering the thoracic cavity through newly formed or pre-existing small perforations in the diaphragm. The exact mechanism of air entry into the thoracic cavity remains unclear but is thought to involve either the passage of air from the peritoneal cavity, potentially via the vagina, uterus, and fallopian tubes, through diaphragmatic defects, or the rupture of endometrial implants on the visceral pleura or lung parenchyma [6].

Our patient presented with a 14-year history of recurrent catamenial pneumothorax and subsequently developed inguinal endometriosis. While the interval between the diagnosis of pelvic endometriosis and the onset of catamenial pneumothorax is typically five to seven years [14], the exact timing of inguinal endometriosis development is less well-documented. Given the recurrent nature of these conditions, even after successful surgical treatment, it is important to maintain long-term follow-up and consider early hormonal therapy, especially in patients who have completed childbearing.

## Conclusions

This case report presents a rare instance of a patient with both inguinal endometriosis and catamenial pneumothorax. The present case underscores the importance of a multidisciplinary approach in the management of patients with extra-pelvic endometriosis. Surgical interventions, including laparoscopy and thoracoscopy, are often necessary for accurate diagnosis and definitive treatment. Given the potential for endometriosis to develop in multiple sites over time, even after successful treatment, long-term follow-up, including consideration of hormonal therapy, is crucial. A multidisciplinary team involving gynecologists, surgeons, and other specialists is essential for the optimal management of patients with rare site endometriosis.
